# Qualitative faecal immunochemical tests (FITs) for diagnosing colorectal cancer in patients with histories of rectal bleeding in primary care: a cohort study

**DOI:** 10.1007/s00384-020-03672-1

**Published:** 2020-06-29

**Authors:** Cecilia Högberg, Ulf Gunnarsson, Olof Cronberg, Hans Thulesius, Mikael Lilja, Stefan Jansson

**Affiliations:** 1 Department of Public Health and Clinical Medicine, Unit of Research, Education and Development Östersund, Umeå University, Umeå, Sweden; 2 Department of Surgical and Perioperative Sciences, Umeå University, Umeå, Sweden; 3 Department of Clinical Sciences, Lund University, Malmö, Sweden; 4 Department of R & D, Region Kronoberg, Växjö, Sweden; 5 Department of Medicine and Optometry, Linnaeus University, Kalmar, Sweden; 6 School of Medical Sciences, University Health Care Research Centre, Örebro University, Örebro, Sweden

**Keywords:** Colorectal neoplasms, Occult blood, Faecal immunochemical tests, Primary health care, Rectal bleeding

## Abstract

**Background:**

Rectal bleeding is considered an alarm symptom for colorectal cancer (CRC) but it is common and mostly caused by benign conditions. Qualitative faecal immunochemical tests (FITs) for occult blood have been used as diagnostic aids for many years in Sweden when CRC is suspected. The study aimed to evaluate the usefulness of FITs requested by primary care physicians for patients with and without histories of rectal bleeding, in the diagnosis of CRC.

**Methods:**

Results of all FITs requested in primary care for symptomatic patients in the Örebro region during 2015 were retrieved. Data on each patient’s history of rectal bleeding was gathered from electronic health records. Patients diagnosed with CRC within 2 years were identified from the Swedish Cancer Register. The analysis focused on three-sample FITs, the customary FIT in Sweden.

**Results:**

A total of 4232 patients provided three-sample FITs. Information about the presence/absence of rectal bleeding was available for 2027 patients, of which 59 were diagnosed with CRC. For 606 patients with the presence of rectal bleeding, the FIT showed sensitivity 96.2%, specificity 60.2%, positive predictive value 9.8% (95% CI 6.1–13.4) and negative predictive value 99.7% (95% CI 99.2–100) for CRC. For 1421 patients without rectal bleeding, the corresponding figures were 100%, 73.6%, 8.3% (95% CI 5.6–10.9) and 100% (95% CI 99.6–100).

**Conclusion:**

The diagnostic performance of a qualitative three-sample FIT provided by symptomatic patients in primary care was similar for those with and without a history of rectal bleeding. FITs seem useful for prioritising patients also with rectal bleeding for further investigation.

## Background

Colorectal cancer (CRC) is the third most common cancer worldwide with over 1.8 million new cases registered in 2018 [1]. The majority of symptomatic patients diagnosed with CRC initially consult primary care [2].

Rectal bleeding is associated with CRC and is considered an alarm symptom [3]. It is also a common symptom in the general population and it is mostly caused by benign conditions such as haemorrhoids or anal fissures [4–6]. In primary care, it can be difficult to determine if a patient’s history of rectal bleeding can be explained by the patient’s haemorrhoids or if it could emanate from CRC and thus, the patient should be referred to secondary care.

Guidelines on CRC recommend that patients with unexplained rectal bleeding should be referred for further examinations, usually sigmoidoscopy or colonoscopy [7–10]. These procedures are resource heavy, unpleasant for the patients and constitute a small risk for morbidity and even mortality [11]. A test to facilitate the selection of patients for colonoscopy with greater certainty than relying on a history of rectal bleeding only would therefore be useful.

Tests for faecal occult blood have been in use for many years for CRC screening in several countries [12]. Guaiac-based tests (haemoccult) are now being replaced by immunochemical faecal occult blood tests (FITs) which are more sensitive and react only to human blood [13–16]. In the UK, FITs are also recommended for use for symptomatic patients without rectal bleeding that do not meet the criteria stated in the “Suspected cancer: recognition and referral” pathway for CRC [17].

In Sweden, faecal occult blood tests have been used as diagnostic aids in clinical practice in primary care as well as in secondary care for many years. There is no national screening programme and faecal occult blood tests are not included in the suspected cancer pathway recommendations. In around 2005, guaiac-based tests were abandoned in favour of qualitative FITs. These FITs can easily be analysed at primary care centres (PCCs); they use a chromatographic technique with pre-set cutoffs and are visually read by identifying coloured lines.

Evidence is growing that FITs are useful as diagnostic tests in primary care before decisions are taken on the referral of symptomatic patients [18–21]. Studies on patients already referred to secondary care have included those with a history of rectal bleeding [22–27]. To our knowledge, no study in primary care has compared FIT results for patients presenting with and without rectal bleeding, respectively.

The aim of this study was to evaluate the usefulness of qualitative FITs requested by primary care physicians (PCPs) for symptomatic patients with and without histories of rectal bleeding, in the diagnosis of CRC.

## Method

We registered the results of all FITs for patients aged ≥ 18 years requested by PCPs from 1 January to 31 December 2015 at all PCCs in the region of Örebro in Sweden (population 290,890 on 1 November 2015). These data were retrieved from the region’s electronic health record system, NCS Cross, used by all PCCs [28]. Samples registered within 14 days of each other were considered as belonging to the same FIT. The date of the FIT was set as the date of the first faecal sample. If more than one FIT had been provided during the year, we registered the first FIT only. The FIT was considered as positive if one or more of the samples tested positive. When a FIT is requested in Sweden, at most PCCs, it is customary to analyse three faecal samples collected from consecutive bowel movements on different days for each FIT (a three-sample FIT). The analyses in this study were focused on cases with three-sample FITs.

The qualitative FIT Actim Fecal Blood was used for the analyses [29]. Instructions on sampling, storage and analysis were issued by the Department of Laboratory Medicine at the Örebro University Hospital and followed by all PCCs in the region. Actim Fecal Blood is an immunochromatographic dipstick test, in which patients collected an expected mass of 10–20 mg of faeces with a sampling stick attached to a cap which was inserted into a tube with 10 ml of buffer solution. The FITs were analysed by laboratory staff at each PCC laboratory, which were all accredited by Swedac (Sweden’s national accreditation body) and supervised by the Department of Laboratory Medicine at the Örebro University Hospital [30]. Tests were visually interpreted by identifying a coloured line for a positive test. Each dipstick had a built-in control line for quality assurance. The cutoff for a positive result was 50 ng haemoglobin/ml of faecal solution corresponding to 25–50 μg haemoglobin/g faeces, and the test remained positive at 500 ng haemoglobin/ml faecal solution, according to the manufacturer’s instruction at the time of the study. Collected samples could be stored for up to 7 days at room temperature before analysis.

For patients that provided FITs, data on the history of rectal bleeding from 1 month before until 1 month after the FIT date was gathered from the electronic health record system through free text search. The electronic search application Medrave was used for this purpose [31]. All paragraphs with words containing “blood” or “bleed” (“blod” or “blöd” in Swedish) were extracted and these were read by one of the authors (CH). Only phrases that explicitly confirmed or denied a history of blood seen in the faeces, in the toilet or on the toilet paper, were registered. For example, phrases about dark or black faeces, phrases stating that there were no defecation problems or that the faeces looked normal, or where patients were uncertain about the presence of blood, were omitted. For quality control of the electronic search result, the health records for all patients diagnosed with colorectal cancer were also read in full and data from these were extracted by one of the authors (SJ). The results from these readings were compared with the electronic search results for the same patients by CH.

Patients diagnosed with CRC within 2 years after their FIT date were identified from the Swedish Cancer Register [32]. The limit of 2 years was chosen as this is the recommended interval for CRC screening in Europe and it has been used in prior primary care studies concerning FITs in symptomatic patients [18, 20, 33, 34]. Also, it seems likely that a patient having CRC but a negative FIT should be diagnosed within 2 years.

### Statistics

SPSS version 24 (IBM, Armonk, NY, USA) was used for statistical analysis. Sensitivity, specificity, positive predictive value (PPV) and negative predictive value (NPV) with 95% confidence intervals (CI), as well as the positive and negative likelihood ratios (LR) of FITs for the diagnosis of CRC were calculated for patients with and without a history of rectal bleeding.

## Results

**Fig. 1:**
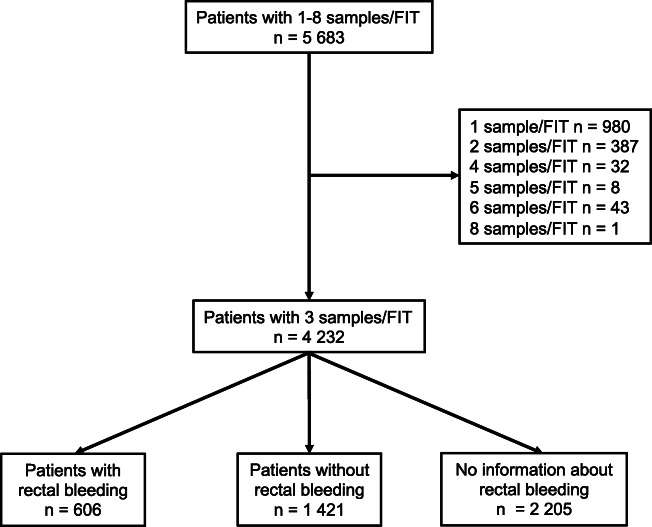
Number of patients that delivered faecal immunochemical tests (FITs) in Örebro region 1 January to 31 December 2015

In total, 5683 (59.9% women) patients at 29 PCCs provided FITs with one to eight samples (Fig. 1). The median age was 64 years (interquartile range [IQR] = 45–76 years). Three-sample FITs were provided by 4232 patients (74.5%; 60.7% women, median age 62 years [IQR = 43–74]). Of all 5683 patients, 107 (1.9%; 43.0% women, median age 75 years [IQR = 66–82]) were diagnosed with CRC within 2 years. Of the 4232 patients that provided three-sample FITs, 79 (1.9%; 45.6% women, median age 73 years [IQR = 64–81]) were diagnosed with CRC (Table 1).

**Table 1:** Qualitative FITs requested in primary care in symptomatic patients, stratified for the number of samples per FIT and related to diagnoses of colorectal cancer

	1–8 samples *n* = 5683	1 sample *n* = 980	2 samples *n* = 387	3 samples *n* = 4232	4 samples *n* = 32	5 samples *n* = 8	6 samples *n* = 43	8 samples *n* = 1
Colorectal cancer *n*	107	19	6	79	2	0	1	0
True positive	102	16	6	77	2	0	1	0
False negative	5	3	0	2	0	0	0	0
False positive	1754	285	187	1230	18	6	28	0
True negative	3822	676	194	2923	12	2	14	1
Sensitivity %	95.3	84.2	100	97.5	100	N/A	100	N/A
Specificity %	68.5	70.3	50.9	70.4	40.0	25.0	33.3	100
PPV % (95%CI)	5.5 (4.5–6.5)	5.3 (2.8–7.9)	3.1 (0.7–5.6)	5.9 (4.6–7.2)	10.0 (0–23.1)	N/A	3.4 (0–10.1)	N/A
NPV % (95%CI)	99.9 (99.8–100)	99.6 (99.1–100)	100 (98.1–100)	99.9 (99.8–100)	100 (73.5–100)	100	100 (76.8–100)	100
LR+	3.03	2.84	2.49	3.29	1.67	N/A	1.5	N/A
LR−	0.07	0.22	0	0.04	0	N/A	0	N/A

*CI*, confidence interval; *FIT*, faecal immunochemical test; *LR+*, positive likelihood ratio; LR−, negative likelihood ratio; *NPV*, negative predictive value; *PPV*, positive predictive value; *N/A*, not applicable

Information about the presence or absence of rectal bleeding was available for 2404 patients, of which 2027 (84.3%; 62.0% women, median age 58 years [IQR = 39–71]) provided three-sample FITs. Of these 2027 patients, 59 (2.9%; 45.8% women, median age 71 years [IQR = 64–80]) were subsequently diagnosed with CRC; 26 with and 33 without rectal bleeding (Table 2). In total, rectal bleeding was registered for 606 (29.9%) of the 2027 patients with three-sample FITs. For patients with a history of rectal bleeding, the sensitivity for CRC of a three-sample FIT was 96.2%, the specificity was 60.2%, the PPV was 9.8% (95% CI 6.1–13.4) and the NPV 99.7% (95% CI 99.2–100). Corresponding values for patients without rectal bleeding were 100%, 73.6%, 8.3% (95% CI 5.6–10.9) and 100% (95% CI 99.6–100) respectively. One patient with a history of rectal bleeding and a negative FIT was diagnosed with CRC. This cancer was registered as ICD-10 C18.1 (malignant neoplasm of the appendix).

**Table 2:** Three-sample qualitative FITs requested in primary care in patients with and without histories of rectal bleeding, related to diagnoses of colorectal cancer

	Three-sample FITs *n* = 2027		
	All patients *n* = 2027	Rectal bleeding *n* = 606	No rectal bleeding *n* = 1421
Colorectal cancer *n*	59	26	33
True positive	58	25	33
False negative	1	1	0
False positive	598	231	367
True negative	1370	349	1021
Sensitivity %	98.3	96.2	100
Specificity %	69.6	60.2	73.6
PPV % (95% CI)	8.8 (6.7–11.0)	9.8 (6.1–13.4)	8.3 (5.6–10.9)
NPV % (95% CI)	99.9 (99.8–100)	99.7 (99.2–100)	100 (99.6–100)
LR+	3.23	2.42	3.79
LR−	0.02	0.06	0

*CI*, confidence interval; *FIT*, faecal immunochemical test; *LR+*, positive likelihood ratio; *LR−*, negative likelihood ratio; *NPV*, negative predictive value; *PPV*, positive predictive value

History of rectal bleeding, irrespective of FIT results, showed a sensitivity of 44.1%, a specificity of 70.5% and a PPV of 4.3% (95% CI 2.7–5.9) for CRC when calculated for the 2027 patients who provided three-sample FITs.

For all patients providing three-sample FITs and diagnosed with CRC, the median time to diagnosis was 76 days (IQR = 48–188). For the 26 patients with rectal bleeding, the median time to diagnosis was 64 days (IQR = 38–169) while for the 33 patients without rectal bleeding, the median time was 89 days (IQR = 58–291).

Contents in the electronic health record entries retrieved with Medrave corresponded to the contents found in the manually scrutinized electronic health records for all the 107 patients diagnosed with CRC. No additional information about rectal bleeding was found when reading the entire texts and no electronically retrieved information was found to be incorrect.

## Discussion

Results of all FITs requested in primary care for symptomatic patients in a Swedish region during 2015 were retrieved, and CRC cases diagnosed within 2 years were identified from the Swedish Cancer Register. The diagnostic performance in the detection of CRC using a qualitative three-sample FIT was similar in patients with a history of rectal bleeding compared with those with no rectal bleeding and was better than that of a history of rectal bleeding alone. This indicates that FITs may be useful for prioritising patients for further investigation also when they have experienced rectal bleeding.

Previous studies with patients referred to secondary care have shown FITs to be superior to English national guidelines (NG12), which include referral of patients aged 50 and over with unexplained rectal bleeding and patients aged 40 and over with unexplained rectal bleeding combined with other symptoms [22, 35]. In the present primary care study, FITs showed a higher sensitivity as well as a higher PPV for CRC than a history of rectal bleeding. This supports the use of FITs, which seem potentially preferable to decision-making based on rectal bleeding only.

This study has some limitations. It is retrospective and information about the presence or absence of rectal bleeding was available for less than half of the patients. It seems probable that PCPs have different habits concerning phrases used in medical records, that patients with information about rectal bleeding may not be equally distributed between PCPs and that the presence or absence of rectal bleeding may be registered more often in patients with more serious symptoms. It is also possible that the presence of rectal bleeding was recorded to a greater extent than the absence of bleeding. On the other hand, in four studies on patients referred to secondary care with the same magnitude of CRC diagnoses as in this study (3.0–5.4%), and where a history of rectal bleeding was registered, the prevalence of rectal bleeding was 24.8–36.0% which is similar to the 29.9% in this study [23, 24, 26, 27]. Another aspect is that only patients for whom FITs were requested and subsequently provided were included in the study, and it is probable that an unknown number of patients with rectal bleeding were referred without providing FITs. However, the study reflects the clinical situation and it seems likely that the PCPs requested FITs when they were in need of a diagnostic aid.

The study also has a number of strengths. It is population-based and data on FITs were collected from electronic health records with complete coverage of the region’s PCCs, including cities as well as rural areas. The electronically retrieved data about rectal bleeding seem reliable, as a comparison of Medrave search results with the manually scrutinized electronic health records for patients diagnosed with CRC revealed no discrepancies. It is unlikely that patients with the occurrence of a CRC diagnosis were missed, as the Swedish Cancer Register has almost total coverage and completeness [36]. Furthermore, the organisation of the public Swedish health care system and the accreditation of the PCC laboratories by Swedac guarantees uniform processing of samples in all PCCs included.

To our knowledge, this is the first study in primary care that evaluates FIT results for patients with a history of rectal bleeding. In a recently published Swedish study, 60 patients referred for colonoscopy had a history of rectal bleeding, and a FIT with a cutoff of > 10 μg Hb/g faeces showed 100% sensitivity, 74.1% specificity, 30.0% PPV and 100% NPV for CRC for these patients [27]. A Scottish study evaluating the accuracy of a quantitative FIT and faecal calprotectin in patients referred for investigation of bowel symptoms, and in which 33.9% of the patients had a documented history of rectal bleeding, showed a 4.3% PPV of rectal bleeding for CRC which is similar to the present study [24]. An English study examining the diagnostic accuracy of a quantitative FIT provided by patients referred to secondary care, in which 36% of the patients had a documented history of rectal bleeding, found that there was only a small difference in the optimal one-sample FIT cutoff value for patients with rectal bleeding versus no bleeding [26].

To conclude, qualitative FITs seem useful for prioritising patients with rectal bleeding in primary care for further investigation. Future prospective studies are desired to further evaluate the accuracy of FITs in patients with rectal bleeding, including the optimal number of samples per FIT and the optimal cutoff value.

## Data Availability

Data is available from the authors upon reasonable request.
